# Unravelling the Link between Polyphenol Intake and the Risk of Digestive System Cancer: An Umbrella Review Using Meta-Analyses and Systematic Reviews

**DOI:** 10.1017/erm.2025.10027

**Published:** 2026-01-08

**Authors:** Arezo Amjadi, Hamid Abbasi, Atefeh Tahavorgar, Mohammadreza Esfahanian, Mahdie Torkaman, Adel Shahrokhi Sardoo, Ali Erfanimanesh, Ali Shamsi-Goushki, Mohammad Esmail Akbari, Barbod Alhouei, Maryam Gholamalizadeh, Saeid Doaei

**Affiliations:** 1 Department of Nutrition, School of Nutritional Sciences and Food Technology, Kermanshah University of Medical Sciences, Kermanshah, Iran; 2 Student Research Committee, Faculty of Nutrition and Food Technology, Shahid Beheshti University of Medical Sciences, Tehran, Iran; 3 School of Nutritional Sciences and Dietetics, Tehran University of Medical Sciences, Tehran, Iran; 4 School of Medicine, Guilan University of Medical Sciences, Rasht, Iran; 5 Department of Chemical Engineering, Science and Research Branch, Islamic Azad University, Tehran, Iran; 6 Department of Nutrition, Faculty of Public Health, Iran University of Medical Sciences, Tehran, Iran; 7 Mashhad University of Medical Sciences, Mashhad, Iran; 8 Cancer Research Center, Shahid Beheshti University of Medical Sciences, Tehran, Iran; 9 Unit of Nutrition and Cancer, Cancer Epidemiology Research Program, Catalan Institute of Oncology, Bellvitge Biomedical Research Institute (IDIBELL), L’Hospitalet de Llobregat, Barcelona, Spain

**Keywords:** dietary intake, digestive system cancer, polyphenol, flavonoid, quercetin

## Abstract

**Background:**

Digestive system cancers (DSCs) constitute a significant number of cancer cases and are closely associated with modifiable risk factors.

**Objective:**

This umbrella review synthesizes evidence from meta-analyses on the association between dietary polyphenol consumption and the risk of DSCs, addressing limitations in the literature and identifying optimal polyphenol types and doses.

**Methods:**

Following Preferred Reporting Items for Systematic and Meta-Analyses (PRISMA) guidelines, a comprehensive literature search was conducted across PubMed, Scopus and Web of Science until April 2025, using specific keywords related to polyphenols and DSCs. Eligible studies included meta-analyses that examined polyphenol intake and DSC risk. The quality was assessed via the AMSTAR 2 and GRADE framework. Statistical analyses were performed using RStudio, employing random-effects models based on the heterogeneity metrics.

**Results:**

Data from six meta-analyses, encompassing 27 effect sizes, revealed a statistically significant 11% reduction in the risk of DSCs associated with polyphenol consumption (RR: 0.89; 95% CI: 0.85–0.93; I^2^: 63%). Subgroup analysis revealed significant risk reductions for specific polyphenol classes: flavonols (22%), quercetin (22%), anthocyanidins (16%), flavan-3-ols (12%) and isoflavones (9%). Publication bias was evident, but adjustments using the trim-and-fill method still indicated a 13% overall reduction in risk (RR: 0.87; 95% CI: 0.83–0.92; I^2^: 64%).

**Conclusions:**

Our findings support the protective role of dietary polyphenols against DSCs, particularly flavonols and quercetin, suggesting that further investigations into the optimal intake levels and mechanisms of action are needed. These findings underscore the potential of dietary modification as a strategy for DSC prevention.

## Introduction

Different parts of the world have a diverse range of cancer epidemiology, but in most places, digestive system cancer (DSC) continues to be a substantial burden on public health systems and a major cause of cancer (Ref. 1). Oesophageal, liver, pancreatic, gallbladder, gastric and colorectal cancers are the most common forms of cancers of the digestive system. The number of new cases and deaths from colon cancer is expected to reach 20 million in 2022, with 9.7 million deaths from the disease in both genders (Ref. 2). Many modifiable variables, such as *Helicobacter pylori* infection, diets high in nitrates and nitrites, salt-preserved foods, tobacco use and alcohol consumption, are associated with an increased incidence and progression of DSCs (Ref. 3, 4).

Dietary intake plays a dual role in the initiation and development of DSCs, including both carcinogenic and protective elements, which can profoundly influence human health and disease progression. Previous studies have indicated that high-polyphenol diets, such as plant-based and Mediterranean diets, may have positive effects on various types of cancer, including DSCs (Refs 5, 6).

Potential mechanisms by which polyphenols prevent cancer include limiting the growth and genesis of tumours by altering the gut microbiota and cancer stem cells, reducing inflammation and exerting antibacterial effects (Ref. 7).

Based on a meta-analysis conducted in 2022, polyphenol consumption decreased the risk of gastric cancer (GC) in both sexes, but to a greater extent in women. However, the study failed to suggest an effective dose for selected polyphenols. Since only observational studies were included, they did not draw a firm conclusion about causation (Ref. 8). Another review in 2024 focused on the mechanism of the antitumour effects of resveratrol against *H. pylori* and reported that resveratrol effectively reduces the proliferation of GC cells. Moreover, resveratrol and chemotherapy/radiotherapy have synergistic effects that may be helpful in therapeutic practice. The authors suggested that more clinical studies on resveratrol intake and GC risk are needed to provide the opportunity to optimize dose selection (Ref. 9). To address this gap, an umbrella review of meta-analyses was designed to provide a more precise picture of the role of polyphenols in DSC prevention.

## Method

### Protocol and registration

This meta-analysis was based on the PRISMA guidelines as the framework for reporting items for meta-analysis. The meta-analysis was registered in PROSPERO as an international database of prospectively registered systematic reviews in health and social care (ID: CRD42025636878).

### Search strategy

A literature search was conducted across three online databases, namely PubMed, Scopus and Web of Science, up to 21 April 2025, using language-independent use of Medical Subject Headings (MeSH), to explore the associations between the intake of different classes of polyphenols and the risk of DSCs. The following keywords were used: (Polyphenols OR Provinols OR Resveratrol OR Tannins OR quercetin OR kaempferol OR myricetin OR flavonoid OR naringenin OR ‘hydroxybenzoic acids’ OR ‘hydroxybenzoic acid’ OR ‘phenolic acids’ OR ‘phenolic acid’ OR lignans OR alkylphenol) AND (‘Stomach Neoplasms’ OR ‘Gastric Cancers’ OR ‘Gastric Neoplasm’ OR ‘Gastric Cancer’ OR ‘Cancer of the Stomach’ OR ‘Stomach Cancers’ OR ‘Gastric Neoplasm’). Furthermore, a manual search of the references from relevant articles and systematic reviews was performed to identify any further pertinent studies. The corresponding author activated the PubMed email alert system to ensure that any newly published meta-analyses or systematic reviews were not missed. The search strategies used in the aforementioned databases are accessible in Supplementary Material 1.

### Eligibility criteria

Studies were included in this study only if they met the following criteria: (1) studies that were designed as systematic reviews and meta-analyses; (2) studies that investigated the association between polyphenol intake and DSC risk. The following papers were excluded from this umbrella review: (1) *in vitro*, *in vivo* and *ex vivo* studies; (2) letters to the editor; (3) case reports and case series; (4) ecological studies; (5) observational and experimental papers; and (6) papers that were not in English. The PICO framework guiding this umbrella review was defined as follows: population (P: individuals diagnosed with DSCs); intervention (I: intake of dietary polyphenols, either total or any subclass (e.g. flavonoids, phenolic acids)); comparison (C: with or without controls); and outcome (O: risk of DSC).

### Study selection

We used the EndNote software to identify and eliminate duplicate articles after compiling the references. Additionally, we conducted a manual screening to ensure that no duplicates were overlooked by the software. Three authors (M.E., B.A. and H.A.) separately reviewed the titles and abstracts of the papers according to the established eligibility criteria and the PICO framework. Each author also assessed the full texts of the papers for compliance with these criteria. Any discrepancies between the reviewers were settled through consultation with the corresponding authors (S.D. and B.A.), ensuring a thorough and unbiased evaluation process.

### Data extraction

Three independent investigators (M.E., B.A. and H.A.) extracted the following data obtained from the included systematic and meta-analyses, using a standardized extraction form encompassing the following parameters: first author; publication year; country; study design; length of study; number of participants; method used to assess polyphenols, food sources, classes of polyphenols and the relative risk (RR)/odds ratio (OR) between the highest and lowest dietary polyphenol intake categories; quality assessment tool; and outcome. Table 1 displays the basic characteristics of the selected papers.

**Table 1.** Meta-analysis and systematic review characteristics

References	Included studies	Location and duration	Participants (*n*)	Age (yrs)	Dietary polyphenols	Quality assessment scale	Findings
Woo and Kim (Ref. 10)	21	South Korea 2001–2013	944	NR	Total flavonoids ^NS^ Flavonols ^S⬇^ Flavan–3-ols ^S⬇^ Anthocyanidins ^S⬇^ Isoflavones ^NS^ Proanthocyanidins ^S⬇^ Quercetin ^S⬇^ Kaempferol ^NS^ Myricetin ^NS^ Hesperidin ^NS^ Naringenin ^NS^	NR	There was a significant association between flavonol intake and stomach cancer risk only in a small number of studies [OR (95%CI) = 0.68 (0.46–0.99)].
Bo et al. (Ref. 11)	15	China 1999–2015	77024	NR	Flavonoid ^NS^	NR	Found no significant association between flavonoid intake and gastric cancer (GC) (OR = 0.88; 95% CI = 0.74–1.04, I2 = 63.6%).
Grosso et al. (Ref. 12)	143	United Kingdom 1999–2016	NR	NR	Flavonols ^S⬇^ Quercetin ^S⬇^ Kaempferol ^NS^ Myricetin ^NS^ Flavones ^NS^ Flavan–3-ols ^NS^ Proanthocyanidins ^NS^ Catechins ^NS^ Flavanones ^NS^ Hesperidin ^NS^ Naringenin ^NS^ Anthocyanins ^NS^ Isoflavones ^NS^ Genistein ^NS^ Daidzein ^NS^ Glycitein ^S⬇^ Lignans ^S⬇^	NOS Medium–high	Prospective studies found no significant findings (RR = 0.70 95% CI = 0.36, 1.37) and among case–control studies (RR = 1.09 95% CI = 0.84, 1.41). There were a few studies showing lower cancer risk for individual flavonols, quercetin and kaempferol, as well as for proanthocyanidins and anthocyanins.
You et al. (Ref. 13)	7	China 2004–2015	596,553	NR	Isoflavones ^NS^	NOS 7.75/9	The highest intake of dietary isoflavones was not significantly associated with GC risk (OR = 0.97, 95% CI = 0.87–1.09).
Yang et al. (Ref. 14)	6	China 2004–2015	505505	NR	Anthocyanins ^NS^	NOS 7.5/9	Anthocyanin consumption and GC risk were not significantly associated (RR 0.92, 95%CI: 0.81–1.04).
Wang et al. (Ref. 15)	31	China 1990–2016	861359	NR	Fermented soy food ^S⬆^ Non-fermented soy food ^S⬇^	NOS 8/9	In order to calculate the risk of GC associated with soy-based food and isoflavone intake, we pooled the relative risks (RRs) for the highest versus the lowest intake categories. However, the results were not statistically significant, suggesting that isoflavone intake may protect against GC (RR = 0.92; 95% CI: 0.79–1.07).
Fagundes et al. (Ref. 8)	19	Brazil 1999–2021	1,197,857	NR	Flavonoids ^S⬇^ Other polyphenols ^S⬇^	NOS 7/9	Polyphenol consumption decreased GC risk by 29% (RR = 0.71; 95% CI: 0.62–0.81), while flavonoid intake reduced GC risk by 28% (RR = 0.72; 95% CI: 0.61–0.85).

### Methodological quality assessment

Methodological rigour was assessed using the Assessment of Multiple Systematic Reviews method 2 (AMSTAR 2) framework by two authors (H.A. and S.D.). This assessment was executed by one author (H.A.) and subsequently reviewed by the corresponding author (S.D.). The AMSTAR 2 instrument comprises 16 questions containing 7 critical questions, prompting evaluators to respond with options such as ‘No’, ‘No Meta-analysis’, ‘Partial Yes’ or ‘Yes’. The cumulative quality derived from the AMSTAR 2 evaluation is classified into four categories: ‘high quality’, ‘moderate quality’, ‘low quality’ and ‘critically low quality’ (Ref. 16).

### Quality assessment of each pooled analysis using GRADE

We classified the quality of evidence for each distinct pooled analysis as ‘high’, ‘moderate’, ‘low’ or ‘very low’ using the Grading of Recommendations, Assessment, Development, and Evaluation (GRADE) approach. All observational studies are originally regarded by the GRADE system as evidence of low quality. Five of the GRADE method’s eight criteria – the risk of bias, inconsistent results across trials, indirectness of evidence, imprecision and publication bias – have the potential to reduce confidence in the accuracy of effect estimates and result in downgrading. A dose–response relationship, a significant effect size free of possible confounders and the conclusion that all plausible residual confounding would further support conclusions regarding exposure impact are the other three criteria that are suggested to improve or upgrade confidence (Ref. 17). Detailed information regarding the GRADE assessment is available in Table 2.

**Table 2.** GRADE summary of findings for polyphenol intake and digestive system cancer risk

Outcome (Type of polyphenol)	Number of studies (Contributing meta-analyses)	Effect (Pooled RR [95% CI])	Initial certainty	Risk of bias (AMSTAR 2)	Inconsistency (I2)	Indirectness	Imprecision	Publication bias	Downgrades	Certainty of evidence	Comments
Flavonoid	4	0.91 [0.79, 1.04]	Low	Very serious (−2)	No concern (0)	No concern	Serious (−1)	Serious (−1)	−4	Very low	Very high risk of bias from included reviews (critically low AMSTAR 2); CI crosses null; funnel plot asymmetry.
Flavonols	2	0.78 [0.66, 0.93]	Low	Very serious (−2)	Serious (−1)	No concern	No concern	Serious (−1)	–4	Very low	Very high risk of bias from included reviews (critically low AMSTAR 2); high heterogeneity (I2 = 85%); funnel plot asymmetry.
Flavan–3-ols	2	0.88 [0.80, 0.97]	Low	Very serious (−2)	No concern (0)	No concern	No concern	Serious (−1)	−3	Very low	Very high risk of bias from included reviews (critically low AMSTAR 2); funnel plot asymmetry.
Anthocyanidins	3	0.84 [0.74, 0.94]	Low	Very serious (−2)	No concern (0)	No concern	No concern	Serious (−1)	−3	Very low	Very high risk of bias from included reviews (critically low AMSTAR 2); funnel plot asymmetry.
Isoflavones	3	0.91 [0.85, 0.97]	Low	Very serious (−2)	No concern (0)	No concern	No concern	Serious (−1)	–3	Very low	Very high risk of bias from included reviews (critically low AMSTAR 2); funnel plot asymmetry.
Proanthocyanidins	2	0.73 [0.48, 1.11]	Low	Very serious (−2)	No concern (0)	No concern	Serious (−1)	Serious (−1)	−4	Very low	Very high risk of bias from included reviews (critically low AMSTAR 2); very wide CI crossing null; funnel plot asymmetry.
Quercetin	2	0.78 [0.68, 0.90]	Low	Very serious (−2)	No concern (0)	No concern	No concern	Serious (−1)	−3	Very low	Very high risk of bias from included reviews (critically low AMSTAR 2); funnel plot asymmetry.
Kaempferol	2	0.99 [0.72, 1.36]	Low	Very serious (−2)	No concern (0)	No concern	Serious (−1)	Serious (−1)	−4	Very low	Very high risk of bias from included reviews (critically low AMSTAR 2); very wide CI crossing null; funnel plot asymmetry.
Myricetin	2	0.92 [0.56, 1.49]	Low	Very serious (−2)	Serious (−1)	No concern	Serious (−1)	Serious (−1)	−5	Very low	Very high risk of bias from included reviews (critically low AMSTAR 2); high heterogeneity (I2 = 76%); very wide CI crossing null; funnel plot asymmetry.
Hesperidin	2	1.11 [0.95, 1.30]	Low	Very serious (−2)	No concern (0)	No concern	Serious (−1)	Serious (−1)	−4	Very low	Very high risk of bias from included reviews (critically low AMSTAR 2); wide CI crossing null; funnel plot asymmetry.
Naringenin	2	1.11 [0.94, 1.30]	Low	Very serious (−2)	No concern (0)	No concern	Serious (−1)	Serious (−1)	−4	Very low	Very high risk of bias from included reviews (critically low AMSTAR 2); wide CI crossing null; funnel plot asymmetry.
Overall polyphenol effect	7	0.89 [0.85, 0.93] (pred. interval: 0.72, 1.09)	Low	Very serious (−2)	No concern (0)	No concern	Serious (−1)	Serious (−1)	−4	Very low	Very high risk of bias from contributing reviews (critically low/low AMSTAR 2); wide predicted interval crossing null; funnel plot asymmetry.

*Note*: Initial Certainty: started at ‘low’ for all outcomes, as the included meta-analyses predominantly synthesize observational studies on dietary intake and cancer risk. Number of Studies (Contributing Meta-Analyses): this column indicates the count of unique systematic reviews or meta-analyses that contributed to the pooled effect estimate for each specific polyphenol type, as detailed in the manuscript’s text and Figure 2A. Risk of Bias (AMSTAR 2): downgraded based on the overall quality of the contributing systematic reviews/meta-analyses as per the AMSTAR 2 framework. Very serious (−2): applied when a ‘critically low’ AMSTAR 2-rated meta-analysis is a contributing study to the specific pooled effect, indicating fundamental methodological flaws. This severe rating from a single contributing review is sufficient to warrant a two-level downgrade in the certainty of evidence for that outcome. Inconsistency (I^2^): downgraded by 1 (serious) if the I^2^ value for the pooled effect was ≥75%, indicating high and unexplained heterogeneity. Otherwise, no downgrade was applied for heterogeneity. Indirectness: not applicable; no specific concerns regarding indirectness of population, intervention, comparator or outcome were identified. Imprecision: downgraded by 1 (serious) if the 95% confidence interval for the pooled effect was wide and/or crossed the null value (RR = 1.0). For the ‘overall polyphenol effect’, the predicted interval (0.72, 1.09) was considered, as it represents the expected range of effects for individual studies and crosses the null. Publication Bias: downgraded by 1 (serious) for all outcomes due to the visual asymmetry observed in the overall funnel plot (Figure 2B), which suggests a potential for selective reporting across the evidence base. Downgrades: the sum of downgrades across the five domains. Certainty of Evidence: classified as high (0 downgrades), moderate (1 downgrade), low (2 downgrades) or very low (3 downgrades).

### Data synthesis and statistical analysis

The reported RRs, accompanied by their respective 95% confidence intervals (CIs), were used to compute the combined effect size. The selection of an appropriate statistical method, a random-effects or fixed-effects model, was informed by the I^2^ and Q statistics (Refs 18, 19). An I^2^ value exceeding 75% was interpreted as indicative of high heterogeneity, whereas an I^2^ value of 40% or lower suggested low heterogeneity (Ref. 20). In accordance with the classes of polyphenols, subgroup analyses were performed to elucidate potential sources of heterogeneity, which included variations among the selected papers. A graphical examination of the funnel plot was carried out to assess the presence of publication bias in the selected papers, utilizing both asymmetrical and symmetrical models. Trim-and-fill analyses were employed to adjust for any identified publication bias. Statistical analyses were conducted via RStudio software, version 2023.03.1, alongside R language programming software, version 4.3.3, with a statistically significant threshold set at P ≤ 0.05.

## Results

### Study selection

**Figure 1.**
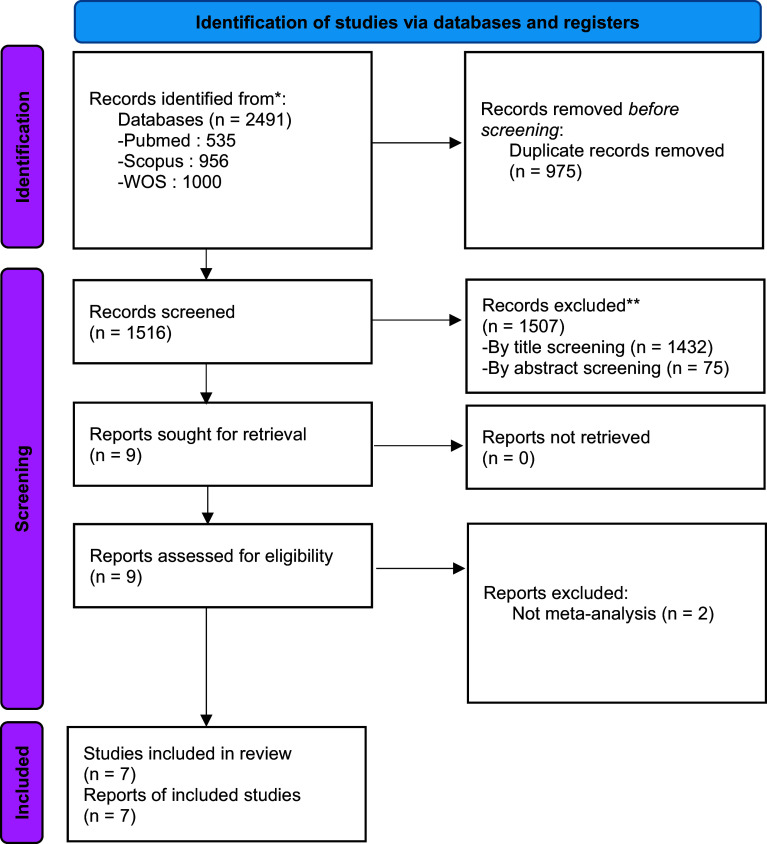
The PRISMA guideline of the study. *Note*: * Consider reporting the number of records identified from each database or register searched (instead of the total number across all databases and registers) if possible. **The number of records excluded by human intervention and the number of records excluded by automation tools, if any, should be listed. *Source*: Page MJ, et al. BMJ 2021;372:n71. doi: 10.1136/bmj.n71. This work is licensed under CC BY 4.0. To view a copy of this license, visit https://creativecommons.org/licenses/by/4.0/

The current research encompasses an umbrella review of meta-analyses published up to 21 April 2025. As shown in Figure 1, a systematic search initially yielded 2491 eligible articles. Following the elimination of duplicate entries, 1516 records were subjected to scrutiny during the title and abstract assessment phase, resulting in the exclusion of 1507 papers. Based on their relevance to the research topic, seven papers were subsequently selected for a comprehensive evaluation of their full texts, with two papers being discarded as they were not meta-analyses.

### Basic characteristics of the selected papers

Table 1 outlines the attributes of the papers included in this umbrella review. The total participant count across these selected meta-analyses was 2,724,242 individuals, with a sample size ranging from 505 to 1,197,857. Among the seven selected meta-analyses, four were conducted in China (Refs 11, 13, 14, 21), whereas one each was carried out in the United Kingdom (Ref. 22), South Korea (Ref. 10) and Brazil (Ref. 8). These meta-analyses investigated the impacts of the consumption of various polyphenols on the odds of developing GC; four studies focused on flavonoids (Refs 8, 11, 22, 10), two focused on flavonols (Refs 22, 10), two focused on flavan-3-ols (Refs 22, 10), three focused on anthocyanidins (Refs 17, 22, 10), three focused on isoflavones (Refs 13, 22, 10), two focused on proanthocyanidins (Refs 22, 10), two focused on quercetin (Refs 22, 10), two focused on kaempferol (Refs 22, 10), two focused on myricetin (Refs 22, 10), two focused on hesperidin (Refs 22, 10), and two focused on naringenin (Refs 22, 10).

### Methodological quality assessment

As shown in Table 3, among the seven chosen meta-studies, one was categorized as having high quality (Ref. 8), two were classified as having low quality (Refs 14, 22), one was deemed to have critically low quality (Ref. 10), and three were assessed as having moderate quality (Refs 11–14).

**Table 3.** Results of the AMSTAR 2 for the selected meta-analyses and systematic reviews

References	Q1	Q2	Q3	Q4	Q5	Q6	Q7	Q8	Q9	Q10	Q11	Q12	Q13	Q14	Q15	Q16	Overall
Woo et al. (Ref. 10)	Y	PY	N	N	Y	N	N	PY	N	N	Y	N	N	Y	Y	N	Critically low
Bo et al. (Ref. 11)	Y	PY	Y	PY	N	Y	PY	Y	PY	N	Y	Y	Y	Y	Y	Y	Moderate
Grosso et al. (Ref. 22)	Y	PY	Y	PY	Y	Y	Y	Y	PY	N	Y	Y	N	Y	Y	Y	Low
You et al. (Ref. 13)	Y	PY	Y	Y	N	Y	PY	Y	PY	N	Y	Y	Y	N	Y	Y	Moderate
Yang et al. (Ref. 14)	Y	PY	Y	PY	N	Y	N	Y	Y	N	Y	Y	Y	Y	Y	N	Low
Wang et al. (Ref. 21)	Y	Y	Y	Y	N	Y	Y	Y	Y	N	Y	Y	Y	Y	Y	N	Moderate
Fagundes et al. (Ref. 8)	Y	Y	Y	Y	Y	Y	Y	Y	Y	N	Y	Y	Y	Y	Y	Y	High

Abbreviations: NOS: Newcastle–Ottawa scale; NR: not reported; NS: non-significant; S: significant; Y: yes; PY: partially yes; N: no; NM: no meta-analysis; questions: Q1: Did the research questions and inclusion criteria for the review include PICO components?? Q2: Did the review report explicitly state that the methods for conducting the review had been established prior to its conduct, and did the report justify significant deviations from the protocol? Q3: How did the review authors decide which study designs to include? Q4: Have the authors conducted a thorough literature search for the review? Q5: Did the review authors select studies in duplicate? Q6: Was data extraction performed in duplicate by the review authors? Q7: Does the review author provide a list of excluded studies and why they were excluded? Q8: Was the description of the included studies adequate by the review authors? Q9: In order to assess the risk of bias (RoB) in individual studies that were included in the review, did the authors use a satisfactory method? Q10: Did the authors of the review report the sources of funding for the included studies? Q11: Is the statistical combination of results used by the review authors if meta-analysis is performed? Q12: Does the review author consider the potential impact of RoB in individual studies on meta-analysis or other evidence synthesis results if meta-analysis was performed? Q13: Was RoB taken into account by the review authors when interpreting/discussing the results? Q14: Did the review authors explain and discuss any heterogeneity observed in the results of the review adequately? Q15: Did the review authors investigate publication bias (small-study bias) and discuss its likely impact on the review results, if they performed quantitative synthesis? Q16: Were there any conflicts of interest reported by the authors, including any funding they received for the review?

### Findings from quantitative analysis

**Figure 2.**
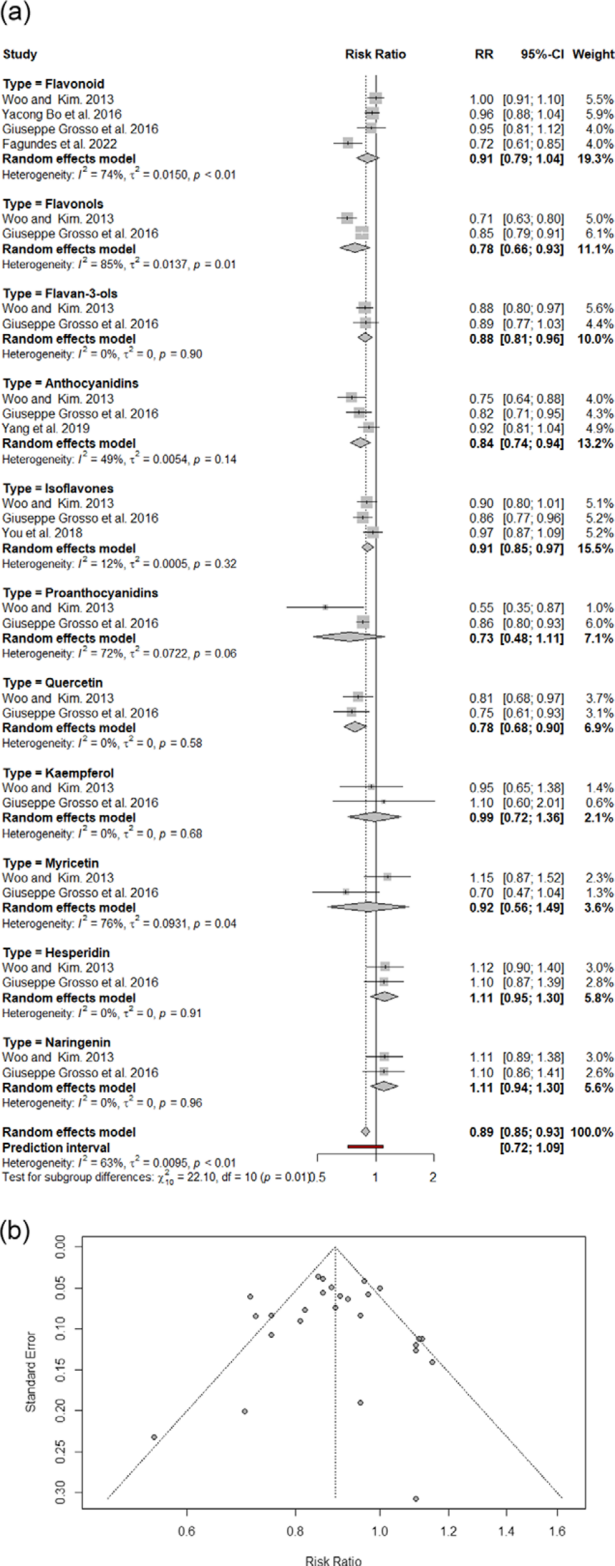
**A**. An overview of meta-analyses and systematic reviews exploring the relationship between dietary polyphenols and digestive system cancer risk; **B**. funnel plot of umbrella review utilizing RR with 95% CI.

The pooled analysis of 27 effect sizes from six meta-analyses revealed that polyphenol consumption was associated with a statistically significant 11% reduction in DSC risk compared with that of controls (RR: 0.89, 95% CI: 0.85, 0.93; I^2^: 63%), with moderate heterogeneity between studies (Figure 2A). A subgroup analysis categorized by polyphenol type revealed meaningful risk reductions of 22% for flavonols, 22% for quercetin, 16% for anthocyanidins, 12% for flavan-3-ols and 9% for isoflavones. The asymmetric funnel plot provided evidence of publication bias (Figure 2B). Following the application of the trim-and-fill method, a significant 13% reduction was detected in the remaining meta-studies (RR: 0.87, 95% CI: 0.83, 0.92; I^2^: 64%).

## Discussion

In the present umbrella review, the results suggested that polyphenol consumption may reduce the risk of DSCs. We found a significant inverse association between the intake of some dietary polyphenol classes, including quercetin, anthocyanidins, flavan-3-ols and isoflavones, and DSCs. Although extensive research in cellular and animal models has explored the chemopreventive effects of polyphenols, epidemiological studies examining the link between polyphenol consumption and specific cancer risks have produced inconsistent results. One possible explanation for this variability is the differing chemical structures and bioactivities of various polyphenol subclasses (Ref. 23).

According to a meta-analysis conducted in 2016, dietary flavonol intake was associated with a reduced risk of GC, as well as between oesophageal cancer and GC combined (Ref. 23). Another meta-analysis revealed no significant associations between the highest dietary flavonoid intake and oesophageal cancer, colorectal cancer or GC. In European populations, higher flavonoid intake is associated with a reduced GC risk (Ref. 11). Anthocyanidins, flavanones and flavones appear to have a protective effect against colorectal cancer. Nimptsch et al. found that the effect only occurred when fruit or vegetable sources of flavonoids were used (Ref. 24). Conversely, there was no association between daily flavonoid intake and colorectal cancer risk in a prospective study of flavonoid intake and colorectal cancer risk (Ref. 25). Additionally, a meta-analysis in 2016 revealed no associations between the incidence of colorectal cancer, and total flavonoids, flavanones and flavan-3-ols, as well as dietary flavonols, flavones and anthocyanidins (Ref. 26). Although another meta-analysis by Lei et al. suggested that there was no significant association, several mechanisms supported by *in vitro* and animal studies remain biologically plausible (Ref. 27). More specifically, *in vitro* studies have shown that flavan-3-ols inhibit cell proliferation and induce apoptosis in colorectal cancer cells (Refs 28, 29, 30).

There have been many studies demonstrating the protective effects of polyphenols in various models, such as quercetin, catechins, isoflavones, lignans, flavanones and ellagic acid. Mechanisms underlying their anticarcinogenic action have been proposed (Ref. 31). For starters, polyphenols may act as blocking agents during the initiation stage of carcinogenesis. They can influence the metabolism of procarcinogens by modulating the expression of cytochrome P450 enzymes that convert them into carcinogens. Additionally, they may promote the excretion of these substances by increasing the expression of phase II conjugating enzymes (Ref. 32). This induction of phase II enzymes may stem from the toxicity of polyphenols, as they can generate potentially harmful quinones in the body, which serve as substrates for these enzymes (Ref. 33). Consequently, the consumption of polyphenols could trigger the activation of these enzymes for detoxification, ultimately strengthening the body’s defences against toxic xenobiotics (Ref. 34). Furthermore, polyphenols may help reduce the formation of initiated cells by stimulating DNA repair processes (Refs 35, 36). Second, polyphenols can function as suppressive agents, inhibiting the formation and growth of tumours from initiated cells and reducing cell proliferation *in vitro* (Refs 30, 37). Research has shown that certain polyphenols can influence growth-related signalling pathways by inhibiting protein kinase C- and AP-1-dependent transcriptional activity (Refs 38, 39). They can also inhibit oncogene expression (Ref. 40) and the activity of ornithine decarboxylase, an essential enzyme involved in the synthesis of polyamines linked to cell proliferation (Refs 41, 42). Furthermore, polyphenols might reduce cell proliferation by affecting the metabolism of arachidonic acid (Ref. 43). Research has shown that dietary flavonol compounds possess multiple bioactivities that can inhibit carcinogenesis and the progression of cancer. These activities include antioxidant, anti-inflammatory, anti-proliferative and anti-angiogenic properties (Refs 44–47). Flavonols may also mitigate the effects of cytokines, growth factors and key enzymes (Refs 48, 49). Quercetin, the primary flavonol found in our daily diets, has been shown to reduce tumour cell viability, promote apoptosis and lower the production of reactive oxygen species (ROS). It achieves these effects by modulating several important signalling pathways, including the IRE1/JNK, PI3K/Akt and FOXO3A pathways (Refs 50, 51).

Quercetin, one of the most abundant dietary polyphenols, exerts its anticancer and protective effects through multiple molecular and cellular pathways. It suppresses inflammatory signalling by inhibiting key pathways such as NF-κB and MAPK, thereby reducing the expression of pro-inflammatory cytokines and mediators (Ref. 52). Moreover, quercetin activates the AMPK/FOXO3a axis and inhibits the PI3K/Akt/mTOR pathway, leading to the suppression of cell proliferation and the induction of apoptosis (Ref. 53). Beyond its potent antioxidant activity, quercetin modulates phase I and phase II detoxifying enzymes, contributing to the detoxification of carcinogens. It also inhibits tyrosine kinase activity and reduces the generation of ROS, thereby preventing DNA damage and mutagenesis. Collectively, these mechanisms highlight the multifaceted nature of quercetin’s bioactivity, emphasizing its regulatory role in (Ref. 54) several key cellular signalling pathways involved in cancer (Ref. 53).

**Figure 3.**
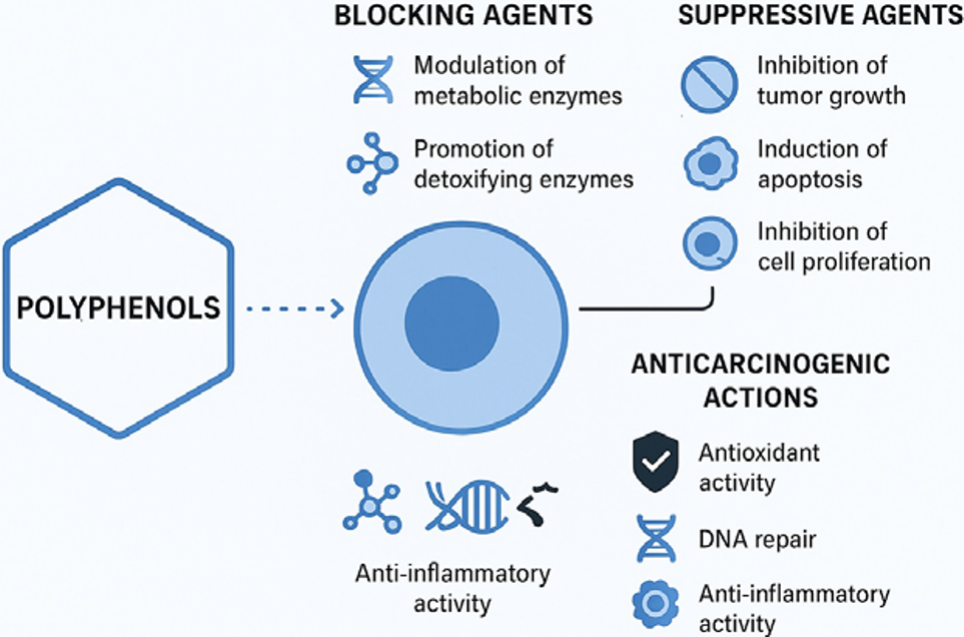
Mechanism of action of polyphenols on cancer.

The role of flavonoids in gastric carcinogenesis is linked to several mechanisms. These compounds exhibit anticarcinogenic effects primarily through their antioxidant properties, which allow them to modulate antioxidant pathways (Ref. 55). Additionally, flavonoids can regulate cell proliferation and apoptosis, influence phase I and II metabolic enzymes and impact inflammatory pathways (Ref. 56). Another explanation for their protective effects could be their activity against *H. pylori* (Ref. 57), including direct bactericidal activity, neutralization of VacA, reduction in urease secretion and interference with Toll-like receptor 4 signalling (Ref. 58), which is particularly relevant to GC. Consequently, a high dietary intake of flavonoids may help reduce the risk of GC, although this effect does not extend to oesophageal or colorectal cancers (Ref. 11) (Figure 3).

### Strengths, weaknesses and future directions

The robustness of this umbrella review is significantly enhanced by its comprehensive and systematic approach. Our adherence to the PRISMA guidelines ensured a meticulous process for selecting and synthesizing relevant literature, providing a broad and rigorous overview of meta-analyses examining the association between polyphenol consumption and the risk of DSCs.

Furthermore, employing a broad search strategy across various databases maximized the capture of pertinent studies, thereby minimizing selection bias. The use of the AMSTAR 2 tool to evaluate the methodological quality of the included meta-analyses further strengthens the review’s credibility, offering a structured and transparent assessment of their rigour. Nevertheless, this umbrella study is not without its limitations, which warrant critical consideration.

First, the diverse methodological quality among the included meta-analyses, with some rated as ‘low’ or ‘critically low’ according to AMSTAR 2, introduces a notable concern regarding the reliability of our synthesized evidence. Lower-quality meta-analyses are inherently more susceptible to biases such as incomplete reporting, inadequate adjustment for confounding variables or methodological flaws in their primary studies. These vulnerabilities can propagate and potentially amplify in an umbrella review, meaning that even significant associations found in our synthesis might be underpinned by less robust original evidence, necessitating a cautious interpretation of pooled effect sizes.

Second, the substantial heterogeneity (I^2^) observed for certain polyphenol classes raises significant concerns about the robustness and direct applicability of our findings across diverse populations. High heterogeneity suggests considerable variability in the true effect sizes across the included meta-analyses, likely stemming from differences in study populations (e.g. genetic variations, lifestyle habits), varied polyphenol assessment methods or inconsistent exposure ranges. Consequently, a single pooled estimate under such conditions may not accurately reflect the effect in any specific context, thereby limiting the generalizability of our quantitative conclusions and hindering the formulation of universal dietary recommendations.

Third, despite the implementation of thorough search strategies aimed at reducing publication bias, there remains a risk that such bias could lead to an inflated estimation of the effect sizes. Additionally, geographical bias is also evident, with a significant number of studies originating from specific regions, particularly China, which may limit the generalizability of the findings to broader populations. Future research should strive to extend the investigation beyond current polyphenol intake methods to foster a more comprehensive understanding of their roles in relation to DSC risks. Moreover, while more included studies adjusted for major confounders, the potential influence of residual confounding variables – such as socioeconomic status, lifestyle, regional dietary habits, comorbidities and genetic predisposition – remains a concern.

Future research should integrate diverse and high-quality epidemiological designs, including large-scale prospective cohort studies across various ethnicities and geographical regions, utilizing detailed dietary assessments and biomarker analyses to explore dose–response relationships and specific polyphenol effects. Furthermore, well-designed randomized controlled trials are critically needed to establish causality, focusing on specific polyphenol interventions, clinical endpoints (including intermediate biomarkers of cancer risk) and mechanistic investigations through ‘omics’ approaches to elucidate precise molecular pathways. Concurrently, deeper exploration of gene–diet and microbiome–diet interactions will help identify responder populations, while the application of advanced statistical methodologies like Mendelian randomization can further minimize residual confounding, ultimately building a more comprehensive and robust understanding of the role of polyphenol intake in DSC prevention.

## Conclusion

In conclusion, this umbrella review highlights the protective role of dietary polyphenols against GC, revealing a significant reduction in the risk associated with increased polyphenol intake. Notably, flavonols and quercetin demonstrated the strongest protective associations. These findings underscore the importance of incorporating polyphenol-rich foods into diets as a potential cancer prevention strategy. Further clinical studies are needed to optimize intake recommendations and explore the underlying mechanisms involved.

## Supporting information

10.1017/erm.2025.10027.sm001 Amjadi et al. supplementary material Amjadi et al. supplementary material

## Supplementary material

The supplementary material for this article can be found at http://doi.org/10.1017/erm.2025.10027.
