# Supplementary material for: Unravelling the Link between Polyphenol Intake and the Risk of Digestive System Cancer: An Umbrella Review Using Meta-Analyses and Systematic Reviews
Source: Expert Rev Mol Med. 2026 Jan 8;28:e3. doi: 10.1017/erm.2025.10027 (PMC12935476; doi:10.1017/erm.2025.10027)
Supplement: Amjadi et al. supplementary material [file S1462399425100276sup001.docx]

**August 6 2024**

| **Database** | **keywords** | **Results number** |
| --- | --- | --- |
| **Pubmed:** | ("Polyphenols"[Mesh] OR Polyphenol*[tiab] OR Provinols[tiab] OR "Resveratrol"[Mesh] OR "Resveratrol"[tiab] OR "Tannins"[Mesh] OR Tannins*[tiab] OR quercetin[tiab] OR kaempferol[tiab] OR myricetin[tiab] OR flavonoid*[tiab] OR naringenin[tiab] OR "hydroxybenzoic acids"[tiab] OR "hydroxybenzoic acid"[tiab] OR "phenolic acids"[tiab] OR "phenolic acid"[tiab] OR lignans[tiab] OR alkylphenol*[tiab])  AND  ("Stomach Neoplasms"[Mesh] OR "Stomach Neoplasms"[tiab] OR “Gastric Cancers”[tiab] OR "Gastric Neoplasm*"[tiab] OR “Gastric Cancer”[tiab] OR “Cancer of the Stomach”[tiab] OR “Stomach Cancers”[tiab] OR “Gastric Neoplasm”[tiab]) | **535** |
| **WOS** | ((((((TS=( polyphenol )) OR TS=(flavonoids)) OR TS=("phenolic acids" )) OR TS=(lignans )) OR TS=(stilbenes )) OR TS=(curcumin )) OR TS=(resveratrol)  AND  (((TS=("gastric adenocarcinoma" )) OR TS=("stomach neoplasms" )) OR TS=("gastric cancer" )) OR TS=("gastric neoplasms" ) | **1000** |
| **Scopus** | ( ( TITLE-ABS-KEY ( polyphenol ) OR TITLE-ABS-KEY ( flavonoids ) OR TITLE-ABS-KEY ( "phenolic acids" ) OR TITLE-ABS-KEY ( lignans ) OR TITLE-ABS-KEY ( stilbenes ) OR TITLE-ABS-KEY ( curcumin ) ) ) AND ( ( TITLE-ABS-KEY ( "gastric adenocarcinoma" ) OR TITLE-ABS-KEY ( "stomach neoplasms" ) OR TITLE-ABS-KEY ( "gastric cancer" ) OR TITLE-ABS-KEY ( "gastric neoplasms" ) ) ) | **956** |
